# The Effect of Pollen Diet Composition and Quantity on Diapause Survival and Performance in an Annual Pollinator (*Bombus Impatiens*)

**DOI:** 10.1093/iob/obad014

**Published:** 2023-04-17

**Authors:** E D Treanore, A V Ramos-Medero, J Garcia, E Amsalem

**Affiliations:** Department of Entomology, Center for Chemical Ecology, Center for Pollinator Research, Huck Institutes of the Life Sciences, Pennsylvania State University, University Park, PA 16802, USA; Department of Entomology, Center for Chemical Ecology, Center for Pollinator Research, Huck Institutes of the Life Sciences, Pennsylvania State University, University Park, PA 16802, USA; Department of Entomology, Center for Chemical Ecology, Center for Pollinator Research, Huck Institutes of the Life Sciences, Pennsylvania State University, University Park, PA 16802, USA; Department of Entomology, Center for Chemical Ecology, Center for Pollinator Research, Huck Institutes of the Life Sciences, Pennsylvania State University, University Park, PA 16802, USA

## Abstract

Most pollination services are provided by annual bees that go through a winter diapause, during which they are exposed to extreme temperatures, pathogens, and starvation. The ability of bees to successfully face these stressors during diapause and subsequently initiate a nest depends on their overall nutritional state and an adequate preparatory diet.

Here, we used queens of the common eastern bumble bee, *Bombus impatiens*, to examine how pollen diets varying in their protein to lipid ratio and total nutrient amounts affected queen performance during and after diapause. We compared diapause survival and reproductive performance post-diapause across different diets and found that queen survival was highest when pollen had a nutritional ratio of approximately 5:1 (protein to lipid). This diet is significantly enriched in proteins compared to the pollen fed to bumble bees in the lab (1:1) or commonly available in agricultural landscapes. Altering the quantity of macronutrients within this ratio did not improve survival or performance. Our results emphasize the importance of adequate nutrition for diapause performance in bees with annual life cycles and the importance of providing annual bees with floral provisioning based on their individual nutritional targets.

## Introduction

Proper nutritional intake is essential for all organisms, ultimately determining the trajectory of development, reproduction, and performance during physiologically demanding periods of the life cycle. Insects have been well studied for their nutritional requirements using the geometric framework, which provides a theoretical foundation to examine how animals forage adaptively for multiple nutrients simultaneously (Raubenheimer and Simpson 1993, 1997, 2003; Vaudo et al. 2016a). By meeting their intake target, e.g., an optimum blend and amount of nutrients, insects can maximize their performance in a given environment through space and time while experiencing fluctuating resource availability (Raubenheimer and Simpson 1997; Simpson et al. 2004). Insects within the same species can have different nutritional requirements depending on their life stage (Runagall-McNaull et al. 2015), sex (Jensen et al. 2015), reproductive state (Jervis et al. 2005), or even when dealing with illness (Povey et al. 2014). Periods of resource dearth or inadequate nutrition can result in a nutrient imbalance (e.g., over ingesting certain nutrients while having deficits in others) that can detrimentally affect an individual's performance (Visser and Ellers 2012; Di Pasquale et al. 2013; Bong et al. 2014; Brunner et al. 2014).

Understanding the consequences of inadequate nutrition is especially important for annual pollinators that undergo a winter diapause, as their survival and performance depend on their ability to regulate their prior nutritional intake. Diapause allows insects to survive inhospitable periods in temperate regions and to synchronize key life history events, such as mating and reproduction, within a population (Tauber and Tauber 1976; Hahn and Denlinger 2011). However, survival of diapause relies on adequate preparation, namely, acquiring and storing sufficient nutrient reserves for meeting an animal's metabolic demands over the winter months (Hahn and Denlinger 2007, 2011). Compromised nutritional intake or the inability to meet the optimum intake target during the diapause preparatory period can result in reduced diapause survival (Galvan et al. 2008; Woodard et al. 2019; Treanore and Amsalem 2020) and may affect post-diapause performance. Most pollination services are provided by annual bees, and many of them are facing population declines, which ultimately pose a threat to global food security (Potts et al. 2010; Van der Sluijs and Vaage 2016). Investigating the impacts of nutrition on diapause survival and performance may provide insight into potential causes of and ways to alleviate population declines.

Bumble bees, and specifically *Bombus impatiens*, provide an excellent system for asking how nutritional intake affects queen performance throughout their life cycle due to their economic importance and the vast knowledge of their biology (Alford 1978; Goulson 2010; Amsalem et al. 2015b). Previous studies show that *B. impatiens* foragers (workers) tightly regulate their nutritional intake around an optimal protein to lipid (P:L) ratio of 5:1 (Vaudo et al. 2016a,b, 2018). Inadequate nutritional intake, such as pollen with a low P:L ratio (Treanore et al. 2019) or that lacks important diet components such as sterols (Moerman et al. 2017) or amino acids (Stabler et al. 2015), can have negative effects on worker reproduction and survival. Furthermore, previous work in *B. terrestris* demonstrated that workers consuming a lipid-enriched diet exhibited reduced survival and fitness (Ruedenauer et al. 2020). However, while these studies have demonstrated the importance of nutrition in mediating worker performance, less is known about the queen's nutritional requirements throughout the colony life cycle. Queens, not workers, found new colonies in the springtime and are the only female caste that survives through the winter (Goulson 2010). Thus, the summer population size of bumble bees is directly impacted by the performance of the queens during diapause. In addition, previous research in bumble bee queens has shown that access to pollen and sugar upon eclosion has long-term effects on adult diapause performance (Woodard et al. 2019), but the composition of nutrients has not been investigated.

Here, we asked if the diet composition queens received prior to diapause affected diapause survival and post-diapause reproductive performance using the bumble bee *B. impatiens*. Specifically, we were interested in whether the ratio of major macronutrients (proteins and lipids) and the amounts of macronutrients within the optimal ratio have long-term effects on queen performance. We examined these questions in two separate experiments. In the first experiment, we compared queen performance on diets skewed toward either lipids or proteins as compared to the common pollen diet provided for bumble bees in captivity (P:L 1:1). In the second experiment, we examined queen performance on diets varying in protein and lipid amounts within a single ratio (P:L 5:1). We hypothesized that queens would perform best around a target ratio and that, within this ratio, either excessive amounts of lipids or proteins would impair queen performance.

## Materials and methods

### Bee rearing

*Bombus impatiens* queens (*n* = 246) were sampled from reproductive-producing colonies (*n* = 9) obtained from Koppert Biological Systems (Howell, MI, USA). Colonies were maintained in a walk-in incubator in the laboratory under constant darkness, 28–30°C, 60% RH, and *ad libitum* access to 60% sugar solution. Fresh honeybee-collected pollen (purchased from Koppert Biological Systems) was provided every other day, also *ad libitum*. Colony maintenance and sampling of individuals were done under a red light to prevent bees from flying.

### Experimental design

Newly emerged queens (determined by a distinct silvery appearance) were separated from their parental colonies upon eclosion and individually tagged with a distinct color and number (Opalith Plättchen). Queens were placed in individual plastic cages (11 cm diameter × 7 cm height) for 7 days and were provided with *ad libitum* sucrose syrup (60%), their assigned pollen diet, and kept under the same conditions as above. The first week following eclosion was shown to be a critical period for nutrient acquisition (Alford 1969; Woodard et al. 2019; Treanore and Amsalem 2020). Following previous work that identified the optimal age range for mating (Treanore et al. 2021), queens were given the chance to mate every day between 7 and 12 days of age (mating conditions described below), and any queens observed mating were placed into cold storage (see below) within 24 h.

In the first experiment, queens were assigned to one of four pollen diet treatments that varied in their protein to lipid ratio (P:L). The treatments were a control diet of ratio 1:1 (pollen collected by honey bees, the most common diet provided to bumble bees in the lab and mass rearing facilities), 1:3 (a diet enriched with lipids), 5:1 (a diet enriched with an intermediate amount of proteins), and 12:1 (a diet enriched with a high amount of proteins). In the second experiment, queens were assigned to one of four pollen diet treatments that varied in the absolute amount of lipids and proteins within the optimal ratio found in the first experiment. The treatments included a control diet of 1:1 P:L and three diets in the ratio of 5:1 with high (×9P and ×1.5L), intermediate (×5P and ×1L), and low (×3P and ×0.5L) amounts of proteins and lipids.

### Diet preparation

Diets were prepared using a combination of pollen collected by honey bees, alpha-cellulose powder (Sigma-Aldrich, St. Louis, MO, USA), casein powder (Sigma-Aldrich, St. Louis, MO, USA), organic flaxseed oil (EarthSource), and DI water. Pollen was ground using a mortar and pestle until it was a fine powder and used as is (100%, “control diet”) or diluted with alpha cellulose to 50% of its original amount (“5:1 low” diet, Exp. 2). Depending on the intended macronutrient ratio and composition of the diet, predetermined amounts of casein powder or flaxseed oil were added (recipes are listed in Table 1). DI water was added to ensure diet texture and appearance were similar across all diets. To verify the total protein and lipid amounts, all diets were analyzed for their macronutrient composition (Table 1). Diet samples that were used for macronutrient composition did not include water.

**Table 1:** The recipe and the amount of proteins and lipids in the diets used in the study.

**Diet (P:L)**	**Pollen (g)**	**Casein (g)**	**Flaxseed oil (g)**	**Cellulose (g)**	**Water (mL**)	**Protein quantity (ug/mg)**	**Lipid quantity (ug/mg)**
1:1 (control)	12	x	x	x	2	45.07 ± 2.62	49.70 ± 2.37
1:3	12	x	2	x	1.5	51.36 ± 1.04	153.91 ± 4.11
5:1 (intermediate)	12	3	x	x	9	228.64 ± 15.02	41.87 ± 2.91
12:1	12	12	x	x	21	518.71 ± 10.55	42.32 ± 2.63
5:1 (high)	12	12	1	x	18	412.36 ± 6.11	64.38 ± 5.67
5:1 (low)	6	12	x	6	26	150.23 ± 8.20	24.50 ± 1.83

### Analysis of diet macronutrients

Lipid and protein amounts were analyzed separately by placing 25 mg of each diet into a sterilized microcentrifuge tube containing 1 mL 2% sodium sulfate (for lipids) and 1 mL 0.1 M sodium hydroxide (for proteins). Samples were homogenized in a fast-prep machine for 45 s and kept at 4°C for a minimum period of 12 h until further analysis.

Lipids were analyzed as in Treanore et al. (2019) and Vaudo et al. (2020) by mixing 200 µL of the homogenate with a 2.8 mL chloroform/methanol solution (v:v 1:1). Samples were centrifuged to achieve separation between the precipitate and the liquid fraction, which was then mixed with 2 mL of distilled H_2_O to separate out the upper lipid fraction. Lipids were quantified by a vanillin-phosphoric acid reaction (5 mL vanillin/sample). A standard curve for lipids was developed using vegetable oil diluted in chloroform.

Protein amounts were analyzed using the Bradford assay (Vaudo et al. 2020) by combining 50 µL of the homogenate with 1500 µL Bradford reagent. A standard curve for protein was developed using bovine serum albumin (BSA) standards (Thermo Fischer Scientific, Waltham, MA, USA) (DeGrandi-Hoffman et al. 2010). Absorbance values (OD 525 for lipids and OD 595 for proteins) were measured using a Synergy LX multi-mode reader (Biotek Instruments, Winooski, VT, USA) and converted to micrograms per 1 mg of pollen based on a formula calculated from a regression line derived from the standard sample values.

### Queen mating

All queens included in the study were mated at the ages of 7–12 days. This range was identified in previous studies as the optimal age for mating in queens (Treanore and Amsalem 2020; Treanore et al. 2021). Once queens reached 7 days of age, they were placed in a mesh arena (60 × 60 × 91 cm) with 2–3 unrelated males for every queen. The mating arena contained 60% sucrose and was placed in ambient light and room temperature for 2–4.5 h per day and observed approximately every 15 min (Treanore et al. 2021). When not in the mating arena, queens were kept in their individual cages to prevent unobserved mating. Any couples observed mating were removed from the mating chamber and placed back into an individual cage until mating ended. Mating pairs remain connected for at least 30 min (Plowright and Jay 1966). Queens that did not mate by the age of 12 days were excluded.

### Cold storage

Queens were weighed and placed into 50 mL Falcon^TM^ tubes with mesh over the top to allow for ventilation within 24 h of mating. Tubes were placed in cardboard boxes (28 × 22 × 5 cm) in conditions simulating diapause (2–4°C, total darkness, and >88% humidity), which we refer to here as cold storage. Survival was checked weekly by exposing queens to room temperature (∼25°C) and ambient light for 10 min and observing them for movement. Queens that displayed movement were put back into cold storage, while queens displaying no movement were marked as dead (Amsalem et al. 2015a; Fauser et al. 2017; Treanore et al. 2020).

### Colony initiation

At the end of a 3-month period, all surviving queens were transferred into individual cages at room temperature (21°C) for approximately 30 min to reduce the stress associated with a rapid change in temperature. Queens were then narcotized with CO_2_ and placed in the walk-in incubator (28–30°C) for another 30 min. While 2 months of cold storage are sufficient to induce a transition to reproduction in *B. impatiens* queens, treating the queens with CO_2_ increases the chance that queens will initiate a colony (Treanore and Amsalem 2022). Queens were treated with CO_2_ using the protocol described in Amsalem and Grozinger (2017). Briefly, a steady stream of pure CO_2_ is applied to a tape-sealed cage containing the queen through a single hole for 1 min. Under these conditions, the cage reaches nearly 100% CO_2_ within seconds, and queens lose mobility within approximately 20 s. The tape is removed after 30 min, during which CO_2_ concentration in the cage is decreased to meet ambient air levels. Queens typically revive within 20 min (Amsalem and Grozinger 2017). Following CO_2_ narcosis, queens were monitored for a period of 3 weeks for survival and egg laying. Queens were placed in individual cages supplied with the control diet (1:1 P:L) to ensure that the outcomes of the study were due to the pre-diapause dietary treatments. Clumps of 4–6 intact pupae collected from full-sized colonies were placed into each cage to stimulate egg-laying behavior. Queen cages were inspected daily for egg cells. Pupae were replaced every 3 days unless they had egg cells built on top of them. The total number of offspring per cage (eggs and larvae) was counted by the end of the 3-week period.

### Statistical analyses

Statistical analyses and data visualizations were performed using R Studio (version 1.4.1106). Generalized linear mixed models (GLMMs) and *lme4* package (Bates et al. 2014) with binomial distributions (yes/no) were used to model queen survival during and post-cold storage. Dietary treatment and mating age were set as fixed effects, and parental colony as a random effect. A GLMM with a binomial distribution (yes/no) was used to model the likelihood of mating pre-cold storage, with treatment as the fixed effect and the parental colony as a random effect. Pre-cold storage mass change was modeled using the *lmer* [*lme4* package] function with treatment set as the fixed effect and colony set as the random effect. Generalized linear models (GLMs) and *glm2* package (Marschner 2011) with binomial distributions (yes/no) and treatment as the fixed effect were used to model the likelihood of egg laying post-cold storage. A GLM with a Poisson distribution and treatment as the fixed effect was used to model the latency to egg laying and number of offspring produced. *P*-values for fixed effects were obtained using the “Anova” (type III) function of the “car” package (Fox et al. 2012). Model selection for each response variable was based on AICc values, biological relevance, and model convergence. Post-hoc tests for all linear models (Tukey post-hoc pairwise comparisons or contrasts between groups) were calculated using the *emmeans* package (Lenth et al. 2018).

## Results

### Mating and pre-cold storage mass gain

The diet treatment did not significantly affect the likelihood of mating in either the first (X^1^_3 _= 5.0162, *P* = 0.17) or the second experiment (X^1^_3 _= 0.78, *P* = 0.85). The average age at mating was 8.18 ± 0.14 and 7.96 ± 0.13 in the first and the second experiment, respectively. In both experiments, queens increased their body mass between eclosion and the onset of cold storage by 20.22 ± 0.01% and 19.0 ± 0.01% on average. The gain in mass of gynes between emergence and the onset of diapause was not significant across diet treatments in the first experiment (X^1^_3 _= 7.49, *P* = 0.06). Queens gained 22.97 ± 0.02% of their mass at emergence when consuming the control diet, 17.93 ± 0.02% in the 1:3 diet, 23.34 ± 0.02% in the 5:1 diet, and 16.32 ± 0.02% in the 12:1 diet. The gain in mass was insignificant across diet treatments also in the second experiment (X^1^_3 _= 2.38, *P* = 0.49). Queens gained 22.31 ± 0.02% in the control diet, 17.53 ± 0.03% in the low diet, 18.46 ± 0.03% in the intermediate diet, and 17.87 ± 0.02% in the high diet.

### Queen survival in cold storage and post-cold storage

**Fig. 1:**
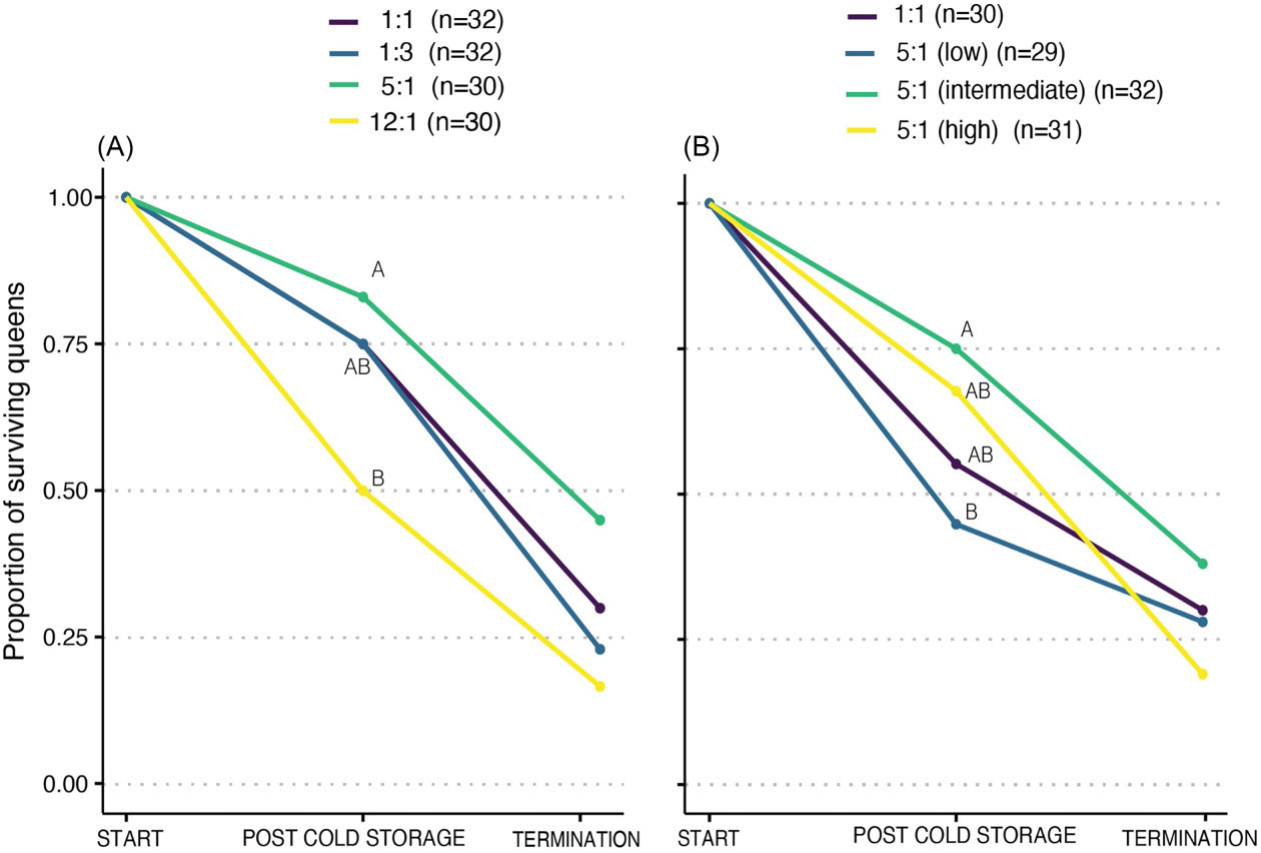
*Bombus impatiens* queen survival during cold storage and in 3-week period following cold storage. Queens were fed pollen diets with different P:L ratios (**A**) and protein and lipid amounts within the 5:1 P:L ratio (**B**). Queens (*n* = 246) were kept in cold storage for a period of 3 months and treated with CO_2_ following their removal from cold storage. Survival was examined at the end of cold storage (“post-cold storage”) and 3 weeks post termination of cold storage (“termination”). Different letters indicate statistical significance at α = 0.05.

Queen survival in cold storage was significantly affected by the pollen P:L ratio in the first experiment (X^1^_3 _= 8.558, *P* = 0.036), with significant post-hoc differences (*P* = 0.04) between queens in the 5:1 (highest survival, 83%) and the 12:1 P:L (lowest survival, 50%) diets. The other two treatments (1:3 and 1:1 P:L diets) were intermediate in their survival rates (75% for both) and did not differ significantly (*P* > 0.05) from the other treatments. Queen survival was also affected by the protein and lipid amounts in the second experiment (X^1^_3 _= 9.169, *P* = 0.027), with reduced survival in the “low” compared to the “intermediate” diet (44% vs. 75%, respectively, *P* = 0.03). The “high” and control diets were intermediate in their survival rates and did not differ statistically from the other treatments. Queen survival in the 3-week period following cold storage was not significantly affected by the pollen P:L ratio (X^1^_3_ = 4.59, *P* = 0.20) or by the amounts of proteins and lipids within the 5:1 ratio (X^1^_3 _= 1.28, *P* = 0.73) (Table 2, Fig. 1).

**Table 2:** The sample size per treatment, the number of queens alive following cold storage and nest initiation, and the number of egg-laying queens in both experiments.

	**Queen alive post-cold storage**	**Queens alive 3 weeks post-cold storage** **	**Egg layers out of initial queens**
**Exp. 1**: Varied macronutrient ratios (P:L)			
1:1 (control)	24/32 (75%)^ab^	10/31 (32%)^a^	10/31 (33%)^ab^
1:3	24/32 (75%)^ab^	7/31 (23%)^a^	8/31 (27%)^ab^
5:1	25/30 (83%)^a^	13/29 (45%)^a^	15/29 (52%)^a^
12:1	15/30 (50%)^b^	5/30 (17%)^a^	4/30 (13%)^b^
**Exp. 2**: Varied macronutrient amounts			
1:1 (control)	17/30 (56%)^ab^	9/30 (30%)^a^	6/30 (17%)^a^
5:1 (low)	13/29 (44%)^b^	8/29 (28%)^a^	6/29 (21%)^a^
5:1 (intermediate)	24/32 (75%)^a^	12/32 (38%)^a^	11/32 (34%)^a^
5:1 (high)	21/31 (67%)^ab^	6/31 (19%)^a^	5/31 (19%)^a^

*Note*: Superscript letters denote statistical significance at α = 0.05.

** Three queens were excluded as they were not treated with CO_2_ following cold storage. These queens were included in the overall sample size of the survival post-cold storage but were removed from any additional analyses.

### Queen reproduction

**Fig. 2:**
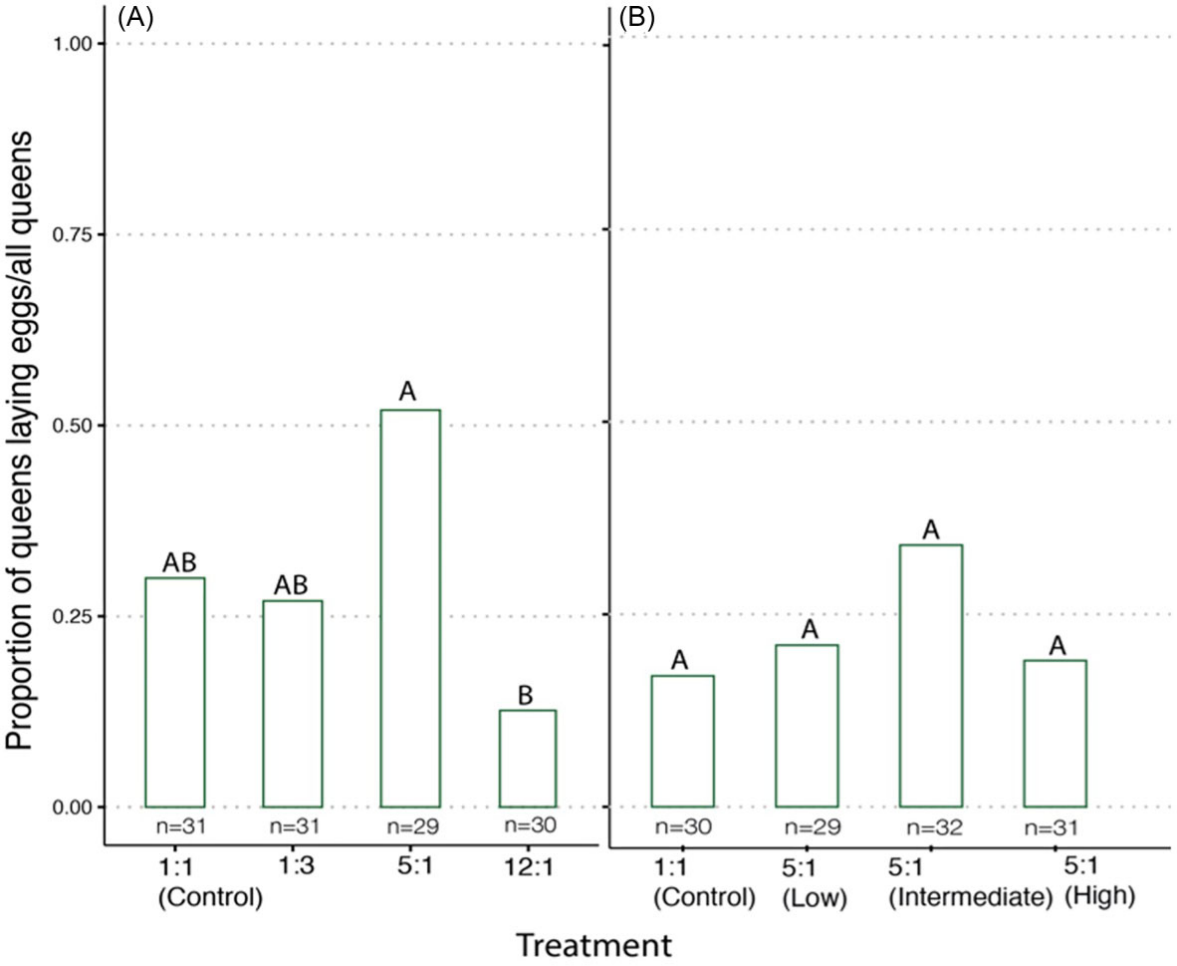
The effect of pre-cold storage P:L ratios (**A**) and protein and lipid amounts (**B**) on the proportion of egg-laying queens. Queens were held in individual cages and were observed for egg laying for 3 weeks following cold storage. The proportions represent the number of egg-laying queens out of the total number of queens placed into cold storage. Different letters indicate statistical significance at α = 0.05.

**Fig. 3:**
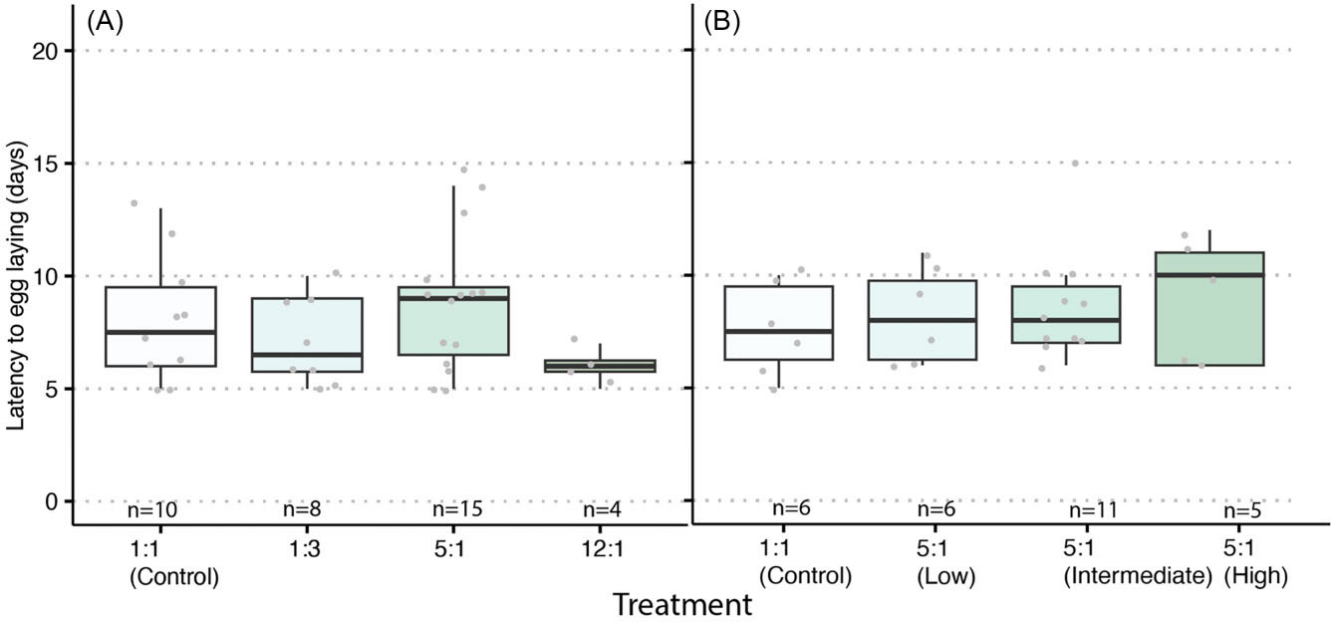
The effect of the pre-cold storage P:L ratios (**A**) and protein and lipid amounts (**B**) on queen latency to egg laying. Queens were held in individual cages and were observed for egg laying for 3 weeks post-cold storage. Boxplots represent the median, first quartile, and third quartile in the data set, and gray dots represent outliers.

**Fig. 4:**
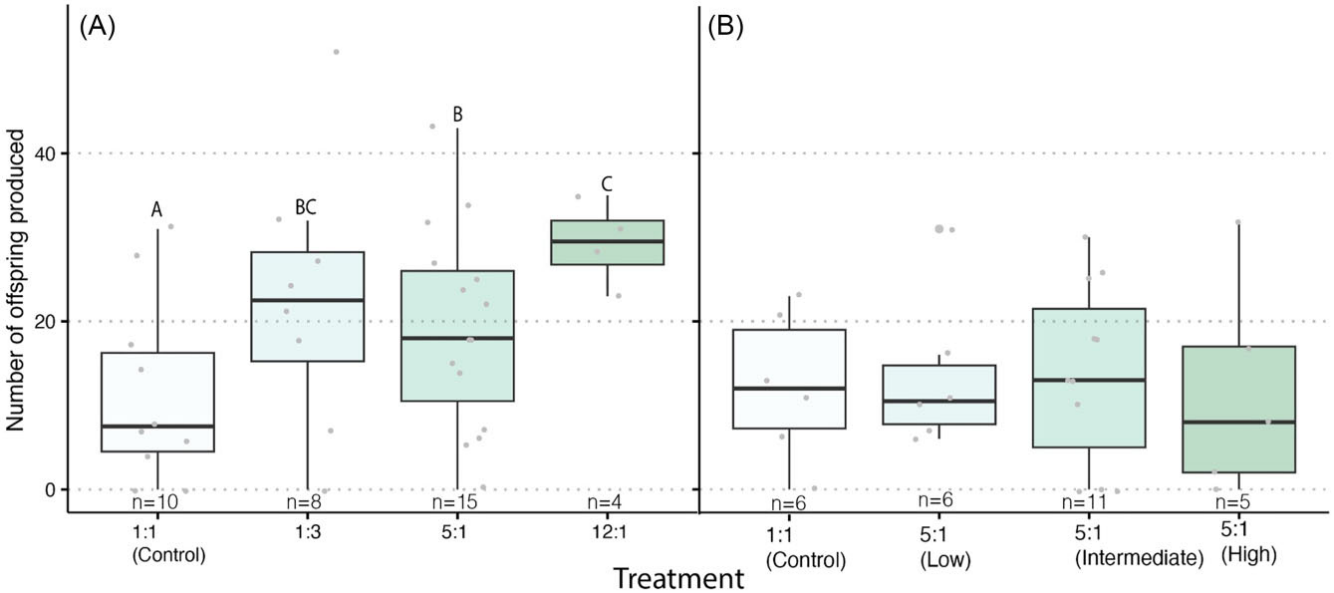
The effect of pre-cold storage P:L ratios (**A**) and protein and lipid amounts (**B**) on the number of offspring (eggs and larvae) produced in a period of 3 weeks post-cold storage. Boxplots represent the median, first quartile, and third quartile in the data set and different letters indicate statistical significance at α = 0.05.

The proportion of egg-laying queens across treatments differed significantly in the first experiment (X^1^_3_ = 10.879, *P* = 0.01) but not in the second experiment (X^1^_3_ = 0.25, *P* = 0.62) (Table 2, Fig. 2). In the first experiment (i.e., different ratios), queens in the 5:1 P:L treatment group were significantly more likely to lay eggs compared to queens in the 12:1 P:L treatment group (*P* = 0.02). No differences were found in the latency to lay eggs in either the first (X^1^_3_ = 4.33, *P* = 0.28) or the second (X^1^_3_ = 0.71, *P* = 0.86) experiment (Fig. 3). The number of offspring produced within the 3-week period was affected by the different P:L ratios in the first experiment (X^1^_3_ = 44.892, *P* < 0.001) with queens consuming the 12:1 diet producing, on average, the greatest number of offspring (29.25 ± 2.53 offspring) and queens in the control group producing, on average, the fewest number of offspring (11.5 ± 3.45 offspring). Different overall amounts of proteins and lipids within the 5:1 P:L ratio did not affect the number of offspring produced (X^1^_3_ = 1.48, *P* = 0.68) (Fig. 4).

## Discussion

Our study demonstrates that pollen diet significantly affects *B. impatiens* queens’ cold storage and post-cold storage performance. Survival was highest in queens that consumed a pollen diet with a macronutrient ratio closest to 5:1, which is also the nutritional intake target for workers (Vaudo et al. 2016a). Similarly, queens consuming diets with intermediate levels of macronutrients had the highest levels of survival during cold storage, suggesting that more proteins and lipids do not necessarily translate to improved survival. However, this did not translate to significantly higher post-cold storage survival, and the effects on queen reproduction were quite varied. Queens consuming the 5:1 P:L diet were significantly more likely to lay eggs compared to queens consuming the 12:1 P:L diet, but the number of offspring produced was significantly higher in the 12:1 diet than all other treatment groups. Our results indicate that proper nutritional intake during sensitive periods of the life cycle can have long-term implications on the survival of an annual pollinator. It also raises interesting questions about the complex effects of dietary protein on survival and reproductive performance, and points to a potential tradeoff queens face when preparing for diapause.

Using cold storage as a proxy, we show clear effects of pre-diapause nutrition on queen performance, with both the ratio and amounts of macronutrients being important. Diapause is known to be a physiologically demanding period of the life cycle (Hahn and Denlinger 2007), and increased mortality will occur if individuals are unable to store sufficient nutrients. Previously, work found that queens placed into cold storage prior to nutrient accumulation (<6 days old) had significantly higher levels of mortality compared to queens that were placed in cold storage later, between 6 and 17 days of age (Treanore and Amsalem 2020). Here, we demonstrate that survival is maximized using pollen with a 5:1 (P:L) ratio, indicating that nutrient availability and quality are both important for an organism entering diapause. This has also been seen in other insect groups, such as the larvae of the khapra beetle (*Trogoderma granarium*), which have decreased survival following consumption of a low-quality pre-diapause diet (determined by dietary freshness) (Shivananjappa et al. 2020). Improved performance was also observed following consumption of intermediate amounts of proteins and lipids within the target ratio. Restriction to a diet that had been reduced to 50% of its original macronutrient amount led to decreased survival, in line with previous work showing that bumble bees do not exhibit compensatory feeding behavior following inadequate nutritional intake (Vanderplanck et al. 2014). Overall, our results highlight how survival can be negatively affected when insects are unable to meet their nutritional needs (Raubenheimer and Simpson 1997).

While cold-storage survival was significantly affected by diet, the effects on reproduction were less pronounced and even contradictory. In the first experiment, the 5:1 P:L ratio resulted in the highest level of survival, and queens were significantly more likely to lay eggs; however, no significant differences were observed between diets that varied in their macronutrient amounts. The latency to egg laying was comparable across almost all the treatment groups, and the dietary ratio with the lowest survival (12:1) had the highest offspring production. One interpretation of these results is that lower-quality queens had reduced survival, but those that survived were of similar reproductive capacity. Alternatively, those that survived were able to replenish their depleted nutrient stores with post-diapause feeding. This has been observed previously in the bruchid, *Kytorhinus sharpianus*, where females displayed similar levels of egg production following post-diapause sugar water feeding (Ishihara and Shimada 1995). The lowest survival and highest offspring production in queens fed on the 12:1 ratio diet can be explained by either a small sample size (only four queens produced eggs in that group) or a tradeoff between survival and reproduction following protein consumption. Notably, a previous study in Drosophila found that females consuming diets with a higher protein to carbohydrate ratio (4:1) had a reduced lifespan but improved egg production (Lee 2015), which mirrors what we observed. But while protein is essential for developing mature eggs, in excess, it may be detrimental due to the high metabolic cost involved in digesting and clearing excess nitrogen (Karowe and Martin 1989; Halton and Hu 2004; Arganda et al. 2017), and the production of potentially toxic waste byproducts (Wright 1995).

Perhaps one of the more interesting results of our study is how queens responded to over- or under-ingestion of protein or lipids as they were limited to a diet with a single nutritional profile that may not have matched their optimal intake target. For example, queens that were restricted to feeding on the most lipid-enriched diet in our study (1:3) demonstrated the sharpest decrease in survival post-cold storage. High dietary lipids have frequently been shown to have detrimental health effects on insect survival, including in *Manduca sexta* larva (Cambron et al. 2019), *B. terrestris* workers (Ruedenauer et al. 2020), and golden-haired blowfly *Calliphora stygia* adults (Ujvari et al. 2009). A previous study on lipid metabolism in insects showed that high dietary lipid amounts may not be taken up quickly enough by midgut cells, resulting in the presence of toxic levels (Canavoso et al. 2001; Ruedenauer et al. 2020). In addition, diets rich in polyunsaturated fatty acids (PUFAs), which are the dominant fatty acids in pollen, can increase the risk of lipid peroxidation in cellular membranes and ultimately reduce survival (Haddad et al. 2007). We also observed that queens limited to the 12:1 P:L diet were the least likely to survive cold storage, indicating that too much protein also had detrimental effects. Similarly, previous work in female *Apis meliffera* (Pirk et al. 2010) and black garden ants, *Lasius niger* (Dussutour and Simpson 2012), showed that imbalanced high-protein diets reduce longevity at both the individual and colony level, suggesting that what we observed is not unique to bumble bees. However, a previous study in *B. terrestris* found that increased amino acids in the diet had little effect on worker survival (Ruedenauer et al. 2020), but this could also result from differences in timescale (21 days in this study vs. >3 months in the current study) or the nature of the manipulation (alteration of specific amino acids vs. general alteration of protein amount). Additional research in Hymenoptera focusing on the effects of dietary protein amounts and quality on survival across life stages could provide further insight into the implications of protein-rich diets.

Overall, our results demonstrate that the pre-diapause pollen diet significantly affects *B. impatiens* diapause survival and post-diapause reproduction and provide insight into the negative effects of over- or under-ingestion of proteins and lipids. This has important implications for both commercial management and improving conservation schemes in the wild. We show that by simply increasing the protein to lipid ratio in the pollen to ∼5:1, the number of queens that reach the point of reproduction following diapause almost doubles—a finding with practical applications for both captive and commercial rearing programs. At present, reared *B. impatiens* queens are frequently fed unaltered honey bee pollen, which typically has a P:L ratio closer to 1:1 (Vaudo et al. 2020). Artificially increasing the amount of protein can bring pollen closer to a 5:1 (P:L) ratio, which, in turn, significantly increase the number of surviving queens. Similarly, ensuring wild bumble bee queens have access to late-summer and fall-flowering floral resources with high P:L ratios can significantly improve diapause survival in wild populations. This can be done through continued characterization of floral resource nutritional profiles (Vaudo et al. 2020) and designing floral provisioning schemes to align with the specific nutritional needs of queens, which may vary between species. Further refining of this approach could be achieved by examining the species-specific nutritional requirements and aligning bee species phenology with habitat enhancement on a regional scale. Future research examining whether nutritional needs shift across life history transitions, i.e., pre-diapause and post-diapause, and whether improved nutrition in later periods of the life cycle can make up for previous dearth, would provide a more holistic perspective on this area of study.

## Data Availability

The datasets generated during and/or analyzed during the current study are available from the corresponding author upon request.
